# SOX4 and RELA Function as Transcriptional Partners to Regulate the Expression of TNF- Responsive Genes in Fibroblast-Like Synoviocytes

**DOI:** 10.3389/fimmu.2022.789349

**Published:** 2022-04-22

**Authors:** Kyle Jones, Sergio Ramirez-Perez, Sean Niu, Umesh Gangishetti, Hicham Drissi, Pallavi Bhattaram

**Affiliations:** ^1^ Department of Orthopaedics, Emory University School of Medicine, Atlanta, GA, United States; ^2^ Department of Cell Biology, Emory University School of Medicine, Atlanta, GA, United States; ^3^ Department of Veterans Affairs, Atlanta VA Medical Center, Decatur, GA, United States

**Keywords:** NF-kappaB, SOX4 transcription factor, RelA/p65, synovial fibroblasts (FLS), TNF, rheumatoid arthritis

## Abstract

SOX4 belongs to the group C of the SOX transcription factor family. It is a critical mediator of tumor necrosis factor alpha (TNF)-induced transformation of fibroblast-like s-ynoviocytes (FLS) in arthritis. In this study we investigated the genome wide association between the DNA binding and transcriptional activities of SOX4 and the NF-kappaB signaling transcription factor RELA/p65 downstream of TNF signaling. We used ChIP-seq assays in mouse FLS to compare the global DNA binding profiles of SOX4 and RELA. RNA-seq of TNF-induced wildtype and *SoxC*-knockout FLS was used to identify the SOX4-dependent and independent aspects of the TNF-regulated transcriptome. We found that SOX4 and RELA physically interact with each other on the chromatin. Interestingly, ChIP-seq assays revealed that 70.4% of SOX4 peak summits were within 50bp of the RELA peak summits suggesting that both proteins bind in close-proximity on regulatory sequences, enabling them to co-operatively regulate gene expression. By integrating the ChIP-seq results with RNA-seq from *SoxC*-knockout FLS we identified a set of TNF-responsive genes that are targets of the RELA-SOX4 transcriptional complex. These TNF-responsive and RELA-SOX4-depenedent genes included inflammation mediators, histone remodeling enzymes and components of the AP-1 signaling pathway. We also identified an autoregulatory mode of *SoxC* gene expression that involves a TNF-mediated switch from RELA binding to SOX4 binding in the 3’ UTR of *Sox4* and *Sox11* genes. In conclusion, our results show that SOX4 and RELA together orchestrate a multimodal regulation of gene expression downstream of TNF signaling. Their interdependent activities play a pivotal role in the transformation of FLS in arthritis and in the inflammatory pathology of diverse tissues where RELA and SOX4 are co-expressed.

## Introduction

Fibroblast-like synoviocytes (FLS) are cells that reside in the synovial lining of joints. During homeostasis, FLS maintain the composition of synovial fluid, produce joint lubricating and cartilage protecting proteins. However, during arthritic diseases they undergo epigenetic changes and transform into pathological cells (1). The transformed FLS are a major source of inflammatory cytokines and catabolic enzymes that promote joint degeneration in autoimmune forms of arthritis such as rheumatoid arthritis (RA), juvenile arthritis, and psoriatic arthritis (1, 2). Among the various signaling pathways that drive FLS transformation, NF-kappaB (NF-κB) signaling downstream of Tumor necrosis factor (TNF)-alpha plays a critical role in joint degeneration by driving a wide range of cellular and molecular changes leading to synovial hyperplasia, cartilage degeneration and bone loss (3, 4). Importantly, the genomic and transcriptomic responses of the FLS from TNF-driven arthritis mouse model are largely comparable to the responses FLS from human RA patients (5, 6). RELA/p65 is the transcription factor that mediates gene expression changes induced by the canonical NF-κB signaling pathway (7). Increase in RELA/p65 protein level and activity were reported in the inflamed synovium of osteoarthritis (OA) and RA patients (8, 9). While p50 is a critical co-factor of RELA for the activation of canonical pathway (10), RELA was also shown to interact with other partners such as p300 acetyl transferase, E2F transcription factor 1(E2F1), Activator Protein 1 (AP1) family in a context dependent manner to activate pro-inflammatory gene expression in a variety of tissues and cells (11–13). In a previous study we showed that, simultaneous conditional deletion of the *SoxC* family (SRY-related HMG-box; Group C) of genes in the FLS inhibits synovitis and articular cartilage degeneration in TNF-induced arthritis in mice (14). The goal of this study is to understand the mechanisms underlying the interaction between TNF and its major transcriptional mediator, RELA/p65 with the SOXC family transcription factors and to determine if SOXC proteins act as transcriptional partners of RELA to promote TNF-induced pathological behavior of the FLS.

SOX4, SOX11, and SOX12 form group C of the SOX family of transcription factors (15). Studies from mouse development, showed that *Sox4*, *Sox11* and *Sox12* are co-expressed in various types of progenitor cells, and act largely in redundancy to determine the behavior and survival of the progenitor cells (15, 16). The SOXC proteins possess a highly conserved DNA binding high mobility group (HMG)-box domain, which enables them to bind to similar SOX binding motifs on DNA. However, they possess divergent transactivation domains in their C-terminus, which confers the highest transactivation potential to SOX11, followed by SOX4. SOX12 possess the weakest transactivation domain (17). As a result, knockout of *Sox12* alone does not affect embryonic development (16, 18). We therefore focused on SOX4 and SOX11 in this study. SOX4 is recognized as a master regulator of cell proliferation and metastasis in several cancer types, while SOX11 recognized as a poor prognosis marker in lymphoma and breast cancer subtypes (19, 20). We previously identified that SOXC proteins, SOX4 and SOX11 play a critical role in inflammation-induced pathological behavior of FLS in osteoarthritis (OA) and RA (14). In addition, *Sox4* and *Sox11* overexpression causes articular cartilage degeneration associated with increased expression of ADAMTS4 and ADAMTS5 (21). At the molecular level, SOXC proteins are highly unstable. They are rapidly degraded under basal conditions but are robustly stabilized upon stimulation with proinflammatory cytokines such as TNF, IL-1 and IL-6 (14). In the current study we used genome wide approaches to uncover the interactions between SOX4 and RELA/p65, downstream of TNF signaling. We thus obtained an in depth understanding of the role and mechanisms underlying the activation of the TNF/SOXC molecular axis in FLS transformation.

## Materials and Methods

### Mice and FLS Preparation

All animal experiments were approved and carried out in accordance with the guidelines by the institutional care and use committee (IACUC) at Emory University. FLS were prepared either from wild type or *Sox4^fl/fl^11^fl/fl^12^fl/fl^* (*SoxC* ^fl/fl^) mice according to a well-established protocol (5, 22). Briefly, fore limbs were separated at the radiocarpal joint while hind limbs were separated at the tibiotalar joint. Skin and nail were removed carefully, and phalanges were separated as to keep the interphalangeal and metacarpophalangeal joints intact. This was followed by a first Collagenase IV (Sigma-Aldrich, 2mg/mL for 30 min) digestion of the isolated joints to remove skin fibroblasts and other surface cells. A second digestion with Collagenase IV (1mg/mL for 2h) was carried out to digest the synovium covering the phalangeal joints. The digests were filtered in a 50-micron cell strainer to remove the undigested bones and debris. The resulting cell suspension containing >90% FLS were cultured in DMEM with 10% fetal bovine serum (Corning) and 1% penicillin/streptomycin for a period of 16h, followed by washing with PBS and changing cell culture medium to remove unattached and dead cells. Upon reaching confluency, the FLS were sub-cultured and utilized between passage 3.

### RNA-seq

RNA-seq was performed in triplicates of independent preparations of FLS. SoxC^fl/fl^ FLS at passage 3 were infected with AdCMV5eGFP (control) or AdCMV5Cre (SoxC-knockout) adenovirus (UI Viral Vector Core) at a concentration of 200 pfu/cell, for a period of 24h. FLS were treated with or without 10ng/mL recombinant human TNF (R&D Systems) for the last 16h. Total RNA was extracted and purified using Direct-zol RNA MicroPrep (Zymo Research). Only samples with an RNA integrity number (RIN) >8 were used. Libraries were generated from 250 ng RNA using TruSeq Stranded Total RNA Sample Prep Kit (Illumina). Paired-end 150-base sequence reads at a sequencing depth of 50 million reads per sample were obtained using Illumina HiSeq 2500 System (Novogene). FASTQ files were analyzed using Strand NGS 4.0 software and aligned to the mm10 mouse genome assembly. Quality of sequencing was ensured by plots for measuring per base sequence quality (Q30 = 99%). Duplicate reads were filtered, followed by quantification and normalization using DESeq method.

### ChIP-seq

Chromatin was prepared and immunoprecipitated according to the following procedure. Triplicates of wild type mouse FLS containing 40 million cells per replicate were treated with or without 10ng/mL TNF for 16 h, followed by fixation with 1.5% paraformaldehyde. Chromatin was extracted and sheared into 100- to 500-bp fragments using a Bioruptor (Diagenode). 10 μg of antibodies against p65 (Active Motif) or SOX4 (Diagenode), coupled to 20 μl Dynabeads (Life Technologies) were used for immunoprecipitating chromatin from each replicate and DNA was purified by phenol/chloroform/isoamyl alcohol extraction and ethanol precipitation. DNA pooled from the triplicates was used for library preparation. Single-end 50-base sequence reads were obtained at a sequencing depth of 30 million reads per sample using Illumina HiSeq 2500 System and analyzed using ChIP-seq pipeline on Strand NGS 4.0 software. Quality of sequencing was ensured by plots for measuring per base sequence quality (Q30 = 97.8%). Reads were mapped to the mm10 mouse genome build. Peak calling and gene annotation was performed using MACS with an upstream padding distance of 50kb. *De novo* motif enrichment analysis was performed using MEME-ChIP suite (23).

### Immunoprecipitation

Wildtype mouse FLS were treated with TNF (10ng/mL) for 16 h. Nuclear extracts were prepared from FLS using NE-PER™ Extraction Reagents (Thermo Scientific) protocol. Immunoprecipitation was carried out by conjugating 5 μg of p65 antibody (Active Motif) or rabbit non-immune IgG (Millipore) to 20 μl Dynabeads (Life Technologies). Cell extracts were added to the conjugated beads overnight and eluted protein was analyzed by western blotting using antibodies against SOX4 (Diagenode), p65 (Cell Signaling) or HDAC1(Cell signaling).

### Luciferase Reporter Assays

ChIP-seq enhancer peak regions assigned to *Sox4*, *Sox11*, *Il15* and *Mapk1* were amplified from mouse genomic DNA and cloned into pBV- Luc, a luciferase reporter plasmid with a minimal promoter and very low basal activity (24). These reporter plasmids were co-transfected with 3X-FLAG SOX4 or 3X-FLAG SOX11 expression plasmids (14) into HEK293 cells using Viromer Red reagent (Origene). pSV2bgal plasmid was used as a control for transfection efficiency. Cells were treated with 10ng/mL recombinant TNF (R&D systems) for the last 16h of the 36h transfection period. Luciferase and β-galactosidase activities were assayed using the Dual-light combined reporter gene assay system (Applied Biosystems) using Synergy H1 Multi-Mode Plate Reader (Biotek). Reporter activities were normalized for transfection efficiency and reported as fold change in luciferase activity. Assays were performed as triplicates.

### Statistical Analysis

Experiments were performed in triplicates. Differential gene expression in changes in RNA-seq were calculated by Audic Claverie and Benjamini Hochberg false discovery rate for multiple testing correction. p-value cut-off of was set at 0.05. Statistical significance in reporter assays was calculated by student’s t-test.

## Results

### SOX4 and RELA Are Transcriptional Partners

**Figure 1:**
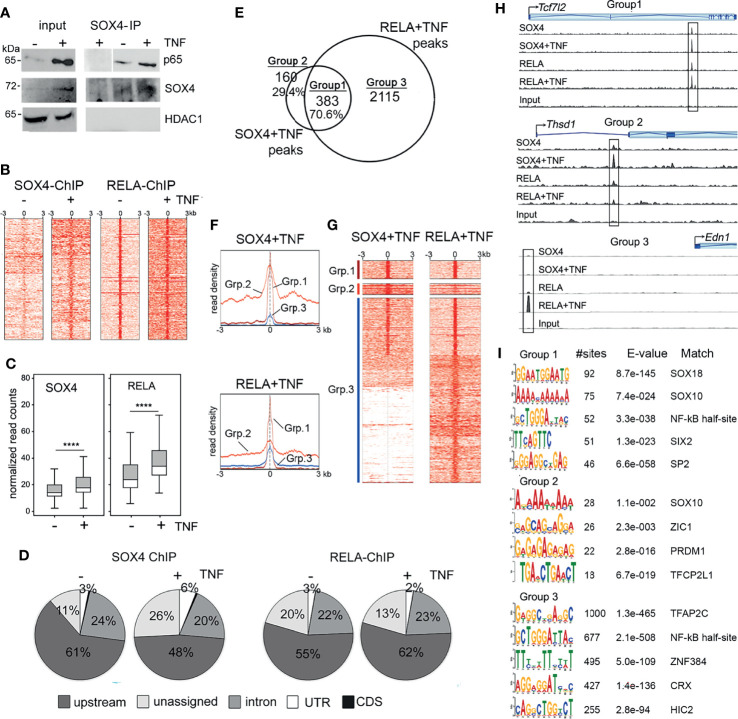
SOX4 and RELA interact on the chromatin **(A)** Immunoprecipitation of nuclear extracts from wildtype FLS treated with or without 10ng/mL TNF for 16 h. Western blot showing lysates and immunoprecipitates detected with the indicated antibodies. **(B)** Heatmap of the read coverage density (more red means more reads at that location) around the transcription start site (TSS) in wildtype FLS treated with or without TNF. **(C)** Box plot representation of normalized read counts in of SOX4 and RELA ChIP-seq peaks. ****p-value < 0.0001 (pairwise t-test adjusted by the Benjamini-Hochberg method). **(D)** Genome wide distribution of SOX4 and RELA ChIP-seq peaks in wildtype FLS treated with or without TNF. **(E)** Venn diagram showing overlap between SOX4 and RELA ChIP-seq peaks that are located within 50bp of each other. **(F)** TSS profile plot and **(G)** Heatmap showing the pattern of binding at the overlapped locations identified by the Venn Diagram in **(F)**. The color bars on the left correspond to Venn Diagram grouping of peaks. **(H)** Genome tracks of SOX4 and RELA ChIP-seq peaks at the loci of representative examples of Group 1, 2 and 3 genes. **(I)** Enriched motifs in the RELA-SOX4 overlapping ChIP-seq peaks in TNF-treated FLS.

We previously showed that the transcription factor SOX4 plays a role in promoting FLS transformation and thereby TNF-induced arthritogenesis (14). To understand the mechanism underlying the role SOX4 in TNF-induced gene expression we investigated whether SOX4 and the canonical NF-κB signaling transcription factor RELA/p65 are part of the same transcriptional complex. We predicted a potential physical interaction between and SOX4 and RELA and tested it by a co-immunoprecipitation assay. We immunoprecipitated RELA from the nuclear extracts of wildtype mouse FLS that were treated with or without TNF (**Figure 1A**). As reported earlier the level of SOX4 protein was increased upon TNF-treatment. Interestingly, we found that SOX4 co-immunoprecipitated with RELA in mouse FLS both in the presence and absence of TNF. However, the interaction was higher under TNF-treated condition likely due to the expected increase in the nuclear localization of RELA. To determine whether SOX4 and RELA interaction occurs at the genome wide level and to identify the genes co-regulated by SOX4 and RELA, we developed an experimental design involving ChIP-seq and RNA-seq assays (**Table 1**). We first performed ChIP-seq using either SOX4 or RELA antibodies. We found that TNF treatment increased the number of SOX4 and RELA binding peaks by 3-fold. The enrichment of sequencing reads from the around the transcription start sites (**Figure 1B**) and box plots (**Figure 1C**) show increased binding of both SOX4 and RELA antibody on the chromatin from TNF-treated FLS in comparison with the untreated FLS. At the genomic level the SOX4 and RELA peaks were predominantly (50-60%) located upstream of TSS. About 20% percent of the peaks were located in the intronic regions. A small percentage (2-6%) of them were localized the 3’ or 5’ untranslated regions and even smaller percentage (>1%) of binding was detected in the coding sequence (**Figure 1D**, **Supplementary Tables S1**–**S4**). Based on the physical interaction between RELA and SOX4 we speculated that these proteins might bind in close proximity to each other at a genome wide level. We overlapped the peak summits identified from SOX4 and RELA ChIP-seq experiments under TNF-treated condition to find that 70.4% of the SOX4 peak summits were present within 50bp of a RELA peak summit which we labelled as Group 1. The peaks with SOX4 only or RELA only peaks were labeled as Groups 2 and 3, respectively (**Figure 1E**, **Supplementary Table S5**). The TSS profile plots and the read density heatmaps show that while read density for Group 1 peaks with SOX4-RELA co-binding is enriched in SOX4 and RELA ChIP-seq and as expected the Groups 2 and 3 peaks show a higher enrichment around TSS only in either SOX4 or RELA peaks respectively (**Figures 1F, G**). The genomic tracks for ChiP-seq peaks at representative examples of Groups 1, 2 and 3 further demonstrate the co-binding and differential binding of SOX4 and RELA (**Figure 1H**). *De novo* motif search by MEME-ChIP discovered SOX binding motifs as the most frequently identified motif in the SOX4-RELA overlapping peaks, followed by a partial NF-κB as reported for CyclinD1 and Il12β genes (25), SIX2 and SP2 binding motifs. Contrastingly motif analysis of the RELA only peaks did not indicate the enrichment of SOX motifs but were instead enriched with AP-2 and NF-kB binding motifs (**Figure 1I**). To determine the functional roles of the genes co-bound by SOX4 and RELA, we compiled a list of 600 genes that were assigned to the RELA-SOX4 overlapping peaks. Network analysis by Ingenuity Pathway Analysis (IPA) revealed a potential crosstalk between the SOX4 and NF-κB signaling regulated genes (**Supplementary Figure S1A**). At the functional level, the SOX4-RELA genes were predicted to regulate pain signaling pathways, xenobiotic stress and tryptophan metabolism, nitric oxide signaling as well as macrophage, fibroblasts, and endothelial cell activities in rheumatoid arthritis (**Supplementary Figure S1B**).

**Table 1:** Conceptual design to study the molecular interactions between RELA and SOXC transcription factors.

Step1: ChIP-seq to identify TNF-induced DNA binding of SOX4 & RELA	
Wild-type mouse FLS treated w or w/o TNF	
SOX4 antibody	RELA/p65 antibody
Identification of SOX4-RELA co-binding and independent DNA binding events	
Step 2: RNA-seq to identify the SOXC and TNF-induced transcriptome	
Sox4^fl/fl^ 11^fl/fl^ 12^fl/fl^ mouse FLS	
AdeCMV5eGFP (control)	AdeCMV5-Cre (SoxC-knockout)
w TNF and w/o TNF	w TNF and w/oTNF
Identification of the TNF-responsive SOXC-dependent gene expression	
Step 3: Classification of TNF-responsive SOXC-dependent genes based on SOX4 and RELA DNA binding			
Integration of ChIP-seq and RNA-seq data			
Class-1	Class-2	Class-3	Class-4
RELA-SOX4 co-binding	RELA only binding	SOX4 only binding	RELA to SOX4 binding switch

### SOX4 Regulates the Expression of TNF-Responsive Transcriptome

**Figure 2:**
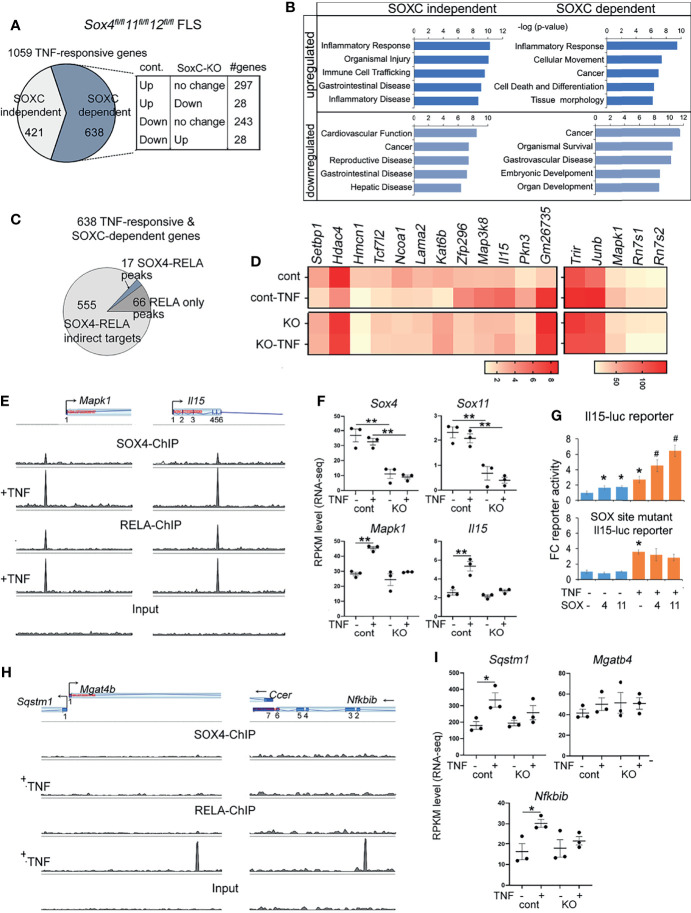
Characterization of SOXC-dependent TNF-responsive genes. **(A)** Pie-chart showing the proportion of SOXC-dependent and SOXC-independent TNF-responsive genes identified from RNA-seq TNF-treated control and SoxC-KO FLS. Cont, *Sox4^flfl^11^flfl^12^flfl^* FLS infected with GFP adenovirus. SoxC-KO, *Sox4^flfl^11^flfl^12^flfl^* FLS infected with GFP adenovirus. The number genes upregulated, downregulated or unchanged by TNF treatment of control and SoxC-KO FLS are indicated in the box. **(B)** IPA pathway analysis of SOXC-dependent and independent TNF-responsive genes. **(C)** Pie chart showing the number of SOX4-RELA co-binding and RELA only bound genes among the SOXC-dependent TNF-responsive genes. **(D)** Heatmap of averaged and normalized RPKM values from RNA-seq of control and SoxC-KO FLS. **(E, H)** Genomic profiles of SOX4 and RELA ChIP-seq peaks. **(F, I)** Gene expression changes by RNA-seq in control and SoxC-KO FLS upon TNF treatment. **(G)** Fold-change *Il15* and SOX-site mutated *Il15* luciferase reporter activity in HEK293 cells transfected with SOX4 or SOX11 expression plasmids and treated with 10ng/mL TNF for 16h. *p-value < 0.05, **p-value < 0.001 by student’s t-test compared to untreated condition. #p-value < 0.05 by student’s t-test compared to SOX4 or SOX11 only conditions.

We next investigated whether SOX4 is required for regulating the global TNF-responsive transcriptome by RNA-seq. We generated *SoxC* knockout FLS by infecting *SoxC* ^fl/fl^ with Cre recombinase-expressing adenovirus. Their corresponding controls were generated by infection with GFP-expressing adenovirus. We utilized total *SoxC* knockout background instead of a *Sox4* knockout background because the *SoxC* family genes (*Sox4, Sox11* and *Sox12*) are functionally redundant and possesses a highly identical HMG-box domain allowing them bind to the same SOX-binding sites on DNA (17). Therefore, deletion of *Sox4* alone may be compensated for by *Sox11* and *Sox12*. We defined TNF-responsive genes as those that were differentially expressed by ≥ 1.5-fold by TNF treatment in the AdeGFP-*SoxC^fl/fl^* FLS. Interestingly, 60% of the TNF-responsive genes remained unchanged or exhibited a reversed regulation in the AdeCre-*SoxC^fl/fl^* FLS indicating that the effect of TNF on FLS is significantly altered in the absence of *SoxC* genes (**Figure 2A**, **Supplementary Figure S2A** and **Supplementary Table S6**). Pathway analysis revealed that that the upregulated TNF-responsive transcriptome which was either SOXC-dependent and independent is predicted to play a role in inflammatory disease pathways, cell migration and tumorigenesis. Interestingly, the downregulated TNF-responsive genes that are SOXC-dependent were predicted to regulate organogenesis and cell survival (**Figure 2B**). We integrated the differential gene expression data with the ChIP-seq results to find that only a small proportion i.e., 17 of 638 *SoxC*-dependent TNF-responsive genes were assigned to RELA-SOX4 peaks and 66 genes were assigned to RELA only peaks (**Figure 2C**). The genes with RELA-SOX4 peaks regulated inflammation mediators such as interleukin-15 (*Il15*), chromatin remodeling factors such as histone deacetylase 4 (*Hdac4*), lysine acetyltransferase 6b (*Katb6*), SET-binding protein 1 (*Setbp1*) and nuclear receptor coactivator 1 *(Ncoa1*) and Activating protein-1 (AP-1) signaling components such as mitogen-activated protein kinase 1 (*Mapk1*/ERK2), mitogen-activated protein kinase 8 (*Map3k8*/MEKK) and JunB proto-oncogene (*Junb*/AP-1) (**Figure 2D**). To understand the clinical significance of the SOXC-dependent TNF-responsive genes we analyzed a published RNA-seq data set from freshly sorted CD45- Podoplanin+ FLS from the synovia from OA and RA patients (26). We found that the expression levels of SOX4, and *SOX11* remained unchanged between the highly inflammatory FLS from leukocyte rich RA patients than in OA patient FLS with low level of inflammation. However, the SoxC-dependent TNF-responsive genes were upregulated in the leukocyte rich RA FLS (**Supplementary Figure S2**). These data suggest that the SOXC/RELA molecular axis may play a critical role in the pathology of highly inflammatory forms of RA.

Using representative examples of SOX4-RELA co-bound genes, such as *Il15* and *Mapk1* we showed the extent of overlap between RELA and SOX4 peaks (**Figure 2E**). Their *SoxC*-dependance was demonstrated by showing that the knockout of *SoxC* genes prevented their TNF-induced upregulation, whereas the levels of *Sox4*, *Sox11* and *Sox12* remained unchanged (**Figure 2F** and **Supplementary Figure S3A**). The *SoxC*-dependent regulation of *Il15* (**Figure 2G**) and Mapk **(** **Supplementary Figure S3B**) were additionally demonstrated in a luciferase reporter assay, where SOX4 and SOX11 overexpression in the presence of TNF significantly increased the activity of a luciferase reporter gene encompassing the SOX4-RELA ChIP-seq peak. Mutation of the two SOXC binding sites in the *Il15* ChIP-seq peak sequence (position 52: AATCAA to AGATCGA and position 168: AAACAAT to AGACAGT) resulted in a loss of SOXC-dependent activation of the Il15-reporter in the absence and presence of TNF. Similarly, examples of genes assigned to RELA only peaks such as Sequestosome 1 (*Sqstm1)*, a ubiquitin binding protein that plays a role in autophagy and NF-κB inhibitor beta (*Nfkbib)*, critical intermediate in the canonical NF-κB signaling were upregulated by TNF only in the presence of *SoxC* genes, but the neighboring gene *Ccer* remained unchanged by TNF or *SoxC* knockout. *Mgatb4* remained unexpressed in the FLS (**Figures 2H, I**).

### TNF Activates a ‘RELA to SOX4 Regulatory Switch’ to Maintain *Sox4* and *Sox11* Gene Expression

**Figure 3:**
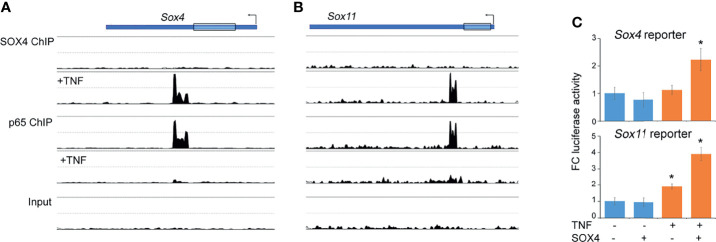
Autoregulatory switch in the *Sox4* and *Sox11* gene expression. **(A, B)** Changes in the profiles of SOX4 and RELA ChIP-seq peaks in the Sox4 and Sox11 genomic locus upon TNF treatment. **(C)** Fold-change Sox4 and Sox11 luciferase reporter activity in HEK293 cells transfected with SOX4 expression plasmids and treated with 10ng/mL TNF for 16h. *p-value < 0.05 by student’s t-test compared to untreated condition.

We previously showed that TNF increases SOX4 and SOX11 protein levels, at least in part by protein stabilization without significantly effecting the mRNA levels (14). Here we addressed the additional mechanisms that might contribute to the TNF-mediated regulation of *Sox4* and *Sox11* expression. In ChIP-seq assays we found that the 3’UTR of *Sox4* and *Sox11* genes were bound by RELA under basal conditions, suggesting that RELA may function as an upstream regulator of *Sox4* and *Sox11* expression. Notably, the RELA binding was lost upon TNF-treatment and this loss corresponded with a gain in SOX4 binding to the same genomic region, suggesting a switch from RELA-mediated expression to an autoregulatory mode of expression (**Figures 3A, B**). We generated luciferase reporter constructs composing the ChIP-seq peak region in the 3’ UTR of *Sox4* and *Sox11* genes to find that a combination of TNF-treatment and transient over expression of SOX4 was necessary for increasing the reporter activity (**Figure 3C**). In consistence with our previous results, TNF-treatment did not result in the level of *Sox4* or *Sox11* expression (**Figure 2F**), indicating that a switch in RELA to SOX4 binding did not alter the overall gene expression, but rather helped in maintaining a consistent level of gene expression in the presence of inflammation.

## Discussion

In this study we utilized genome wide ChIP-seq and RNA-seq approaches to show that (1) The SOXC family member, SOX4 interacts with RELA in FLS. (2) The SOX4-RELA interaction is likely fortified by the proximity of SOX4 and RELA binding sites on the chromatin. (3) A significant subset of the TNF-responsive RELA target genes require *SoxC* genes for their optimal expression. (4) TNF activates an autoregulatory switch, which results in shift from RELA binding to SOX4 binding at the Sox4 and Sox11 regulatory regions.

**Figure 4:**
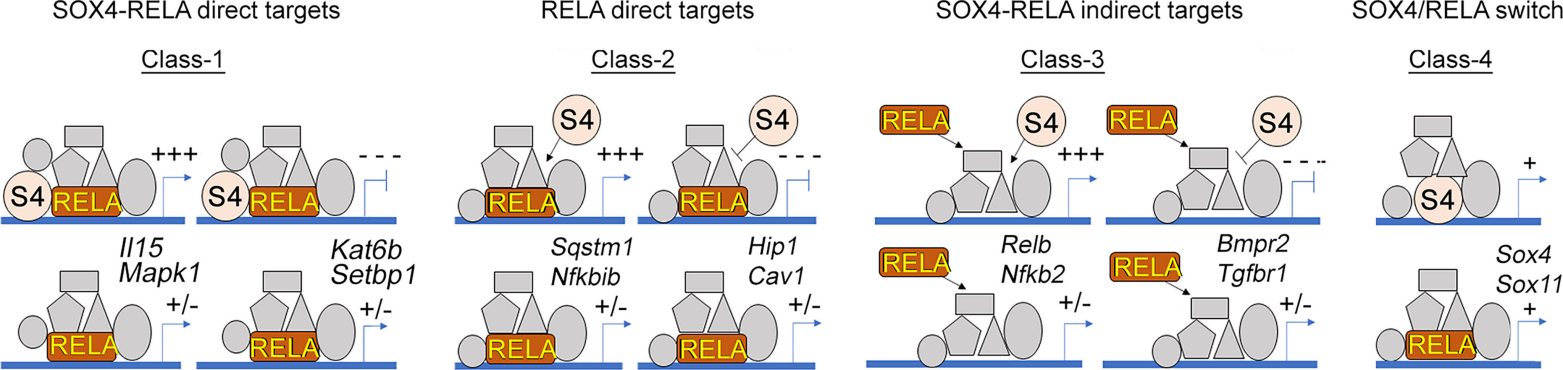
Multimodal regulation of gene expression by RELA and SOX4. Illustration of the 4 different classes of SOXC-dependent and TNF-responsive genes and their predicted responses upon SoxC-knockout are indicated. Two representative examples of each class are indicated.

*SoxC* genes regulate the expression of TNF target genes *via* multiple different modes of action (**Figure 4**); Class-1 includes SOX4-RELA direct target genes that require the binding of both SOX4 and RELA to their regulatory sequences. The Class-2 genes are RELA-direct targets that require only RELA to bind to their regulatory regions, while SOX4 may regulate the expression of one or more of the transcriptional co-factors or upstream regulators of NF-κB signaling. The Class-3 genes are indirect targets of both RELA and SOX4. They are neither bound by RELA or SOX4 and likely use a different transcription factor, whose expression or activity is controlled by RELA and SOX4. The Class-4 genes exhibit regulation *via* ‘regulatory switch’ in which TNF-induces a switch from RELA to SOX4 binding. The autoregulation of *Sox4* and *Sox11* is mediated by this regulatory switch.

We made an interesting finding that about three quarter of all SOX4 binding events induced by TNF (383 out of 584 peaks), were in proximity of a RELA binding event. However, RNA-seq results showed that most of the SOX4-RELA or RELA binding events did not lead to differential gene expression. This suggests that additional factors or stimuli are needed for their expression. Based on the *de novo* binding site predictions by MEME-ChIP, we speculate that these additional factors may include SIX2, SP-2 and AP family of transcription factors. The bulk of SOXC-dependent TNF-responsive transcriptome is constituted by Class-3 genes that are the indirect targets of SOX4 and RELA. Only a small subset of (16 out of 638 genes) belong to Class-1 that require RELA-SOX4 co-binding for their differential regulation. None-the-less the Class-1 genes possess the potential to induce drastic changes in the FLS transcriptome. For instance, the downregulation of histone methylation writer, *Kat6b* and upregulation of histone acetylation eraser, *Hdac4* could potentially remodel the chromatin landscape in the FLS. Overexpression of HDAC4 histone deacetylase correlates with decreased production of proinflammatory cytokines in FLS (27) and decreased expression of Runx2, MMP-13 and collagen X in chondrocytes (28). *HDAC4* was also found to be downregulated in RA synovium (29), which may explain why inflammatory mediator production is actively amplified in RA.

Similarly, differential expression of AP-1 signaling pathway components including *Mapk1* (ERK2), *Map3k8* (MEKK8) and *Junb* (AP1) is also expected to contribute to large-scale cellular and molecular changes. Regarding ERK2, Wang et al. reported that overexpression of this kinase was involved in maintaining cartilage homeostasis through modulating the TGF-β/SMAD axis *via* reducing the expression of *Col2a1*, Aggrecan, and *Sox9*, which are key molecules for cartilage homeostasis (30). The AP-1 subunit, JunB, has been proposed as a negative regulator of proliferation and production of cytokines in fibroblasts (31, 32). Thus, upregulation of *Mapk1* and *Junb* in *SoxC*-KO FLS could indicate a regulatory role for SOXC in inflammatory arthritis.

Our analysis of published RNA-seq data from freshly isolated FLS from leukocyte rich RA patient synovium and OA patient synovium (26) showed a differential regulation of SOXC and TNF-dependent genes, suggesting that the higher level of inflammation present in leukocyte rich RA activates the SOXC/RELA pathway. However, these data did not allow us to comment on the role of SOXC/RELA axis in OA patient FLS, since normal (non-arthritic) FLS were not included in this study. We previously showed that pro-inflammatory cytokines stabilize the protein level of SOX4 and SOX11 and promote inflammatory gene expression in both OA and RA FLS. Supporting this notion, other studies reported a role for SOX4 in promoting the inflammatory phenotype of both OA and RA FLS (33, 34). Taken together, these suggest that the SOXC/RELA axis is activated by inflammation, irrespective of the disease etiology and may thus contribute to the pathology of both OA and RA.

Studies from breast cancer cells showed that SOX4 is a downstream of Transforming growth factor beta (TGFβ) signaling and that SOX4 expression was required for TGFβ-mediated induction of a subset of SMAD3/SOX4-co-bound genes (35). Here we show that SOX4 is required for the induction of RELA/SOX4 co-bound genes, however these co-bound regions did not contain SMAD binding sites. Studies performed in chondrocytes have shown increased expression of ADAMTS-5 after TNF induction in a SOX4-dependent manner (21, 36). These data therefore put forth a notion that SOX4’s choice of transcriptional partners is highly context dependent.

Autoregulation is a mechanism by which a transcription factor regulates its own expression. It is a common mechanism observed in several developmentally important transcription factors to ensure their abundance and activity is not repressed by other factors (37, 38). Several members of SOX family such as *SOX2* and *SOX9* (39, 40) that are master regulators of embryonic development exhibit autoregulation. We here show that *Sox4* autoregulates its own expression in addition to its group-member *Sox11 via* binding to a regulatory region in the 3’ UTR. This finding suggests that SOX4 is critical factor in the regulation of inflammatory responses and hence acquired the property of autoregulation. Interestingly, it was reported that single nucleotide polymorphism (SNP) in the untranslated region (UTR) lead to osteoporosis susceptibility, suggesting an important role for the 3’ UTR in the regulation of SOX4 gene expression in other diseases associated with inflammation (41).

A limitation of this study it presents a simplified view of the TNF/SOXC molecular axis. The functionally redundant activities of *Sox4*, *Sox11* and *Sox12* in TNF-induced arthritis and the diverse range of stimuli that can potentially activate the canonical NF-κB signaling in the FLS are suggestive of a role for multiple additional co-factors besides SOXC and RELA. The proposed Class 1 to 4 modes of interaction between RELA and SOXC proteins (**Figure 4**), especially the TNF-induced autoregulatory switch described in Class-4, requires additional follow up studies to completely unravel its biological significance. Another limitation is the lack of investigation on the role of *Sox12*, the third member of the *SoxC* gene family. Although SOX12 has the weakest transactivation domain among the SOXC proteins (17) it was shown to play a role in the inflammatory response of T cells (42, 43) and cancer cells (44–46). Thus, it may contribute to the transcriptional regulation of inflammation in the FLS, which needs to be determined in future studies. In addition, future investigations using a variety of *in vivo* arthritis models are required to further delineate the molecular and functional interactions of SOXC and RELA proteins during synovial inflammation.

In conclusion, we uncovered the molecular mechanism by which *SoxC* genes regulate the inflammatory responses in the FLS at the genomic level. Our data will serve as a resource for studies on RELA and SOX4 target genes in the FLS arthritic diseases. Together with our previous findings, we demonstrate a role of TNF/SOXC molecular axis in FLS during arthritis and suggest its role in the inflammatory pathology of other cell types where SOXC proteins play a vital role.

## Data Availability Statement

The original contributions presented in the study are publicly available. This data can be found here: NCBI, GSE197694.

## Ethics Statement

The animal study was reviewed and approved by IACUC Emory University.

## Author Contributions

KJ, SR-P, SN, and UG contributed to the generation of the data, data analysis and critical reading of the manuscript. KJ, SR-P, HD, and PB contributed to manuscript writing. PB and HD contributed to study design. All authors contributed to the article and approved the submitted version.

## Funding

We thank the support from the National Institutes of Health/National Institute of Arthritis, Musculoskeletal and Skin Disease grant (R01 AR070736) and Startup funds from the Department of Orthopaedics, Emory University School of Medicine to PB.

## Conflict of Interest

The authors declare that the research was conducted in the absence of any commercial or financial relationships that could be construed as a potential conflict of interest.

## Publisher’s Note

All claims expressed in this article are solely those of the authors and do not necessarily represent those of their affiliated organizations, or those of the publisher, the editors and the reviewers. Any product that may be evaluated in this article, or claim that may be made by its manufacturer, is not guaranteed or endorsed by the publisher.
