# Supplementary material for: SOX4 and RELA Function as Transcriptional Partners to Regulate the Expression of TNF- Responsive Genes in Fibroblast-Like Synoviocytes
Source: Front Immunol. 2022 Apr 22;13:789349. doi: 10.3389/fimmu.2022.789349 (PMC9074688; doi:10.3389/fimmu.2022.789349)
Supplement: Supplementary file 1 [file DataSheet_1.pdf]

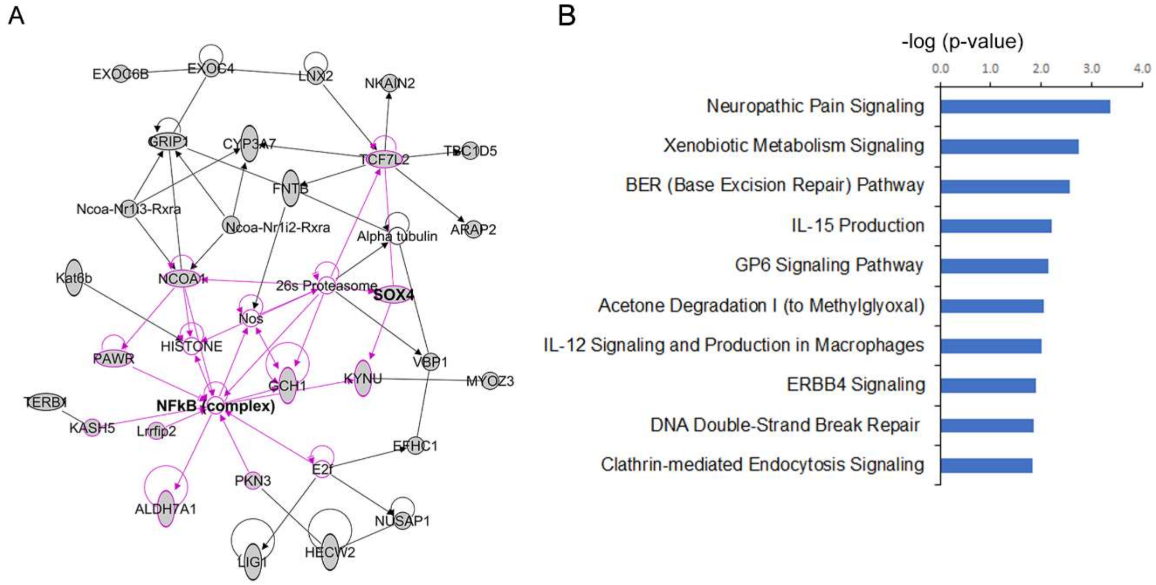

Supplementary Figure S1. Characterization of RELA and SOX4 ChIP-seq peaks. (A) Protein interaction network of RELA-SOX4 co-binding genes by Ingenuity Pathway Analysis. (B) Canonical pathways prediction of RELA-SOX4 co-binding genes by Ingenuity Pathway Analysis.

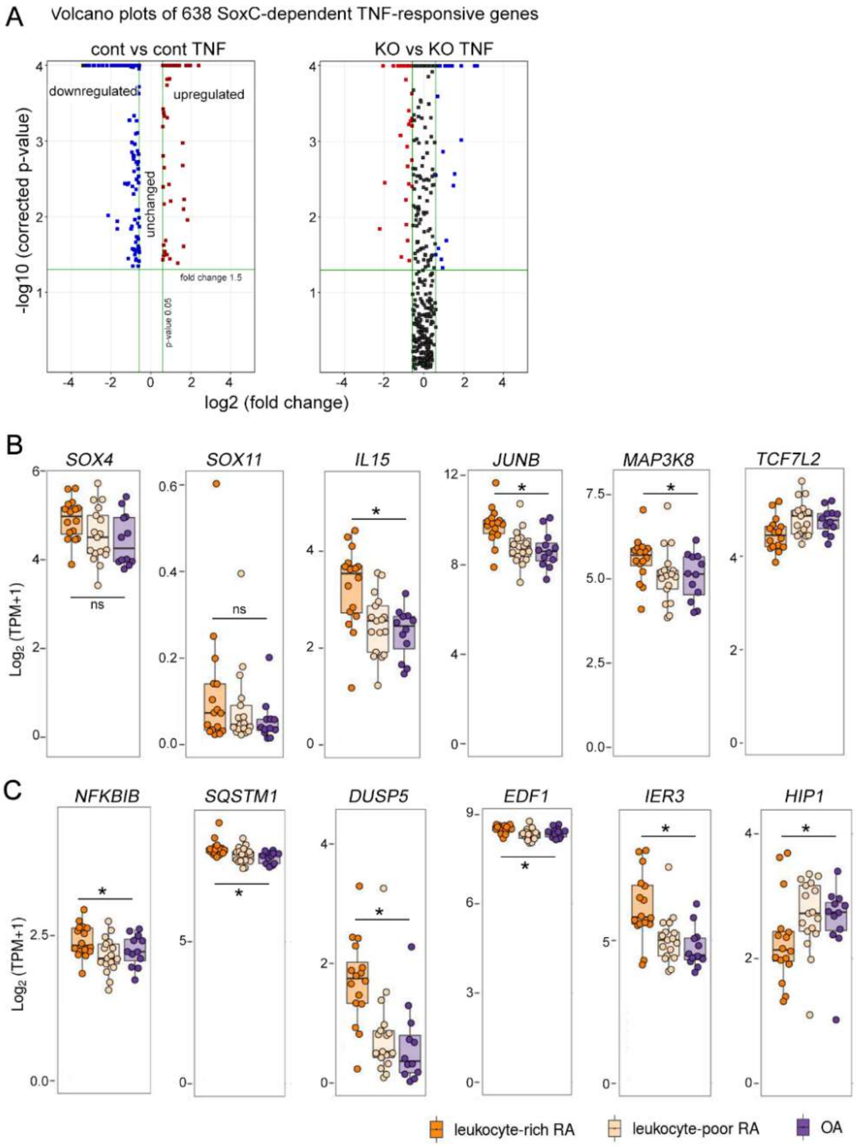

Supplementary Figure S2. Characterization of SoxC-dependent TNF-responsive genes. (A) Volcano plots of RNA-seq data showing comparison differential expression of SoxC-dependent TNF-responsive genes in AdeGFP SoxCfl/fl (cont) FLS and AdeCre SoxCfl/fl (SoxC KO) FLS. Note that the genes significantly upregulated or downregulated by TNF in the cont. FLS are not changed or show a reversed regulation in expression in SoxC knockout FLS. (B and C) Differential expressed genes in publicly available bulk RNA-seq data set from freshly sorted CD45- PDPN+ FLS from RA and OA patients. Data visualized from Accelerating Medicines Partnership (AMP) Rheumatoid Arthritis (RA) Phase I project (https://immunogenomics.io/ampra/). *, p-value less than 0.05 by Kruskal-Wallis test.

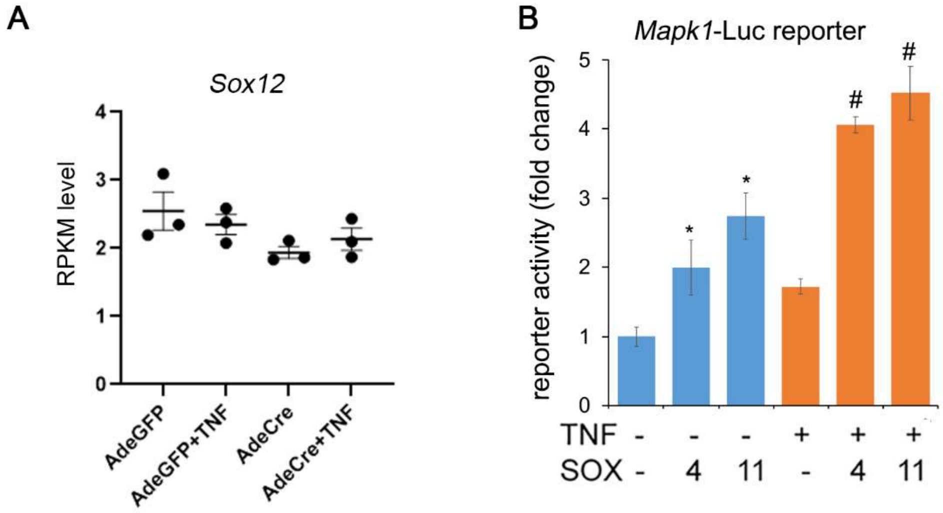

Supplementary Figure S3. (**A**) Changes in *Sox12* gene expression by RNA-seq in control and SoxC-KO FLS upon TNF treatment. (**B**) Fold-change MapI1 luciferase reporter activity in HEK293 cells transfected with SOX4 or SOX11 expression plasmids and treated with 10ng/mL TNF for 16h. * p-value < 0.05 by student's t-test compared to untreated condition. #, p-value < 0.05 by student's t-test compared to SOX4 or SOX11 only conditions.
